# Desquamative extravasation reaction secondary to enfortumab vedotin

**DOI:** 10.1016/j.jdcr.2024.02.003

**Published:** 2024-02-13

**Authors:** Akshay N. Pulavarty, Shane Meehan, Jason Weed

**Affiliations:** a Department of Internal Medicine, New York University Grossman School of Medicine, New York, New York; b The Ronald O. Perelman Department of Dermatology, New York University Grossman School of Medicine, New York, New York

**Keywords:** cancer, desquamation, enfortumab vedotin, extravasation, urothelial carcinoma

## Case report

**Fig 1:**
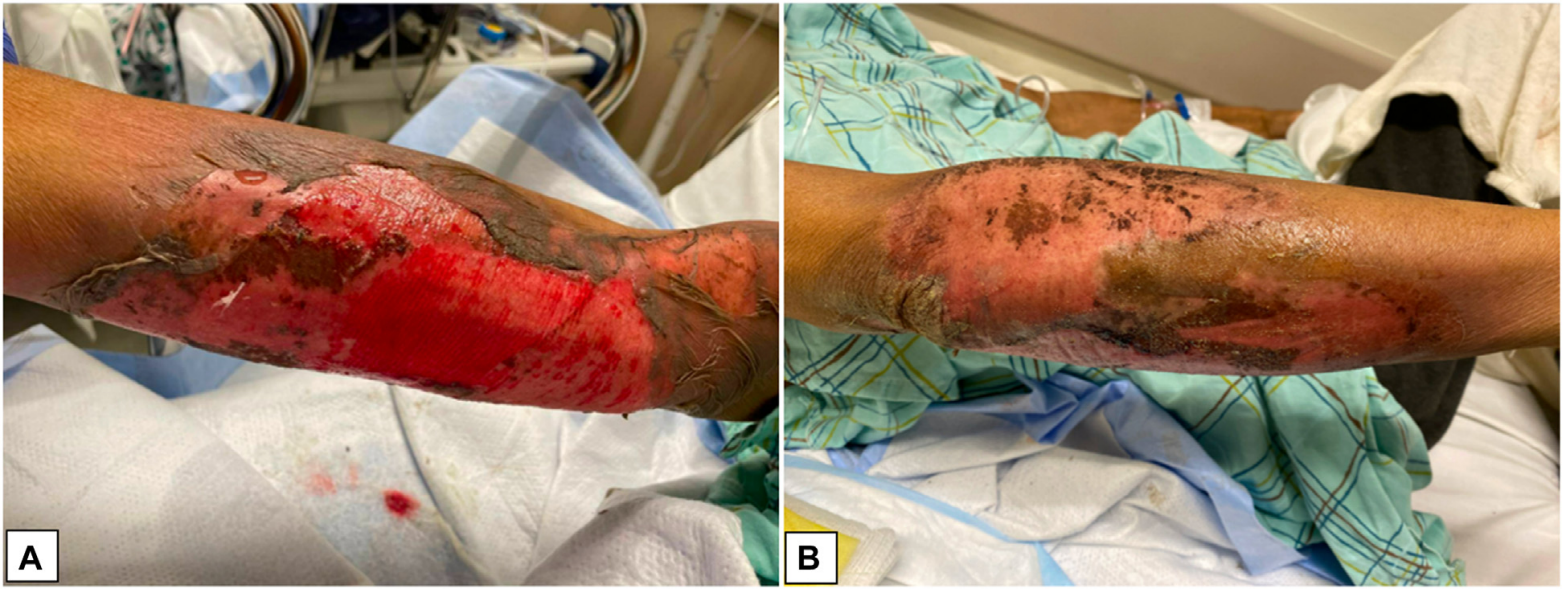
Epidermal sloughing of right upper and mid arm (**A**) extension onto dorsal aspect of the forearm (**B)**, and exposure of the papillary dermis.

A 59-year-old man presented to our hospital with a desquamating rash of the right arm (Fig 1, *A*, *B*). The patient’s history was notable for metastatic urothelial carcinoma. The patient began palliative chemo-immunotherapy with pembrolizumab and enfortumab vedotin. Pembrolizumab and enfortumab vedotin were initially infused through a peripheral intravenous line in the right antecubital fossa, and over the week following the first infusion, he progressively developed epidermal sloughing of the mid-upper arm and forearm (Fig 1, *A*, *B*). On examination, pulses were intact and there was minimal tenderness to palpation. The remainder of the cutaneous examination was unremarkable, and there were no ocular or oral mucosal erosions. Over the subsequent days, his skin findings remained stable and his wounds were dressed with bismuth-petrolatum gauze and gauze wrap which were changed daily.

## Discussion

Cutaneous reactions to antineoplastic therapy vary greatly with one type resulting from direct contact of the extravasated chemotherapeutic. Chemotherapy extravasation has a prevalence of 0.1% to 6% when administered through a peripheral intravenous catheter and can cause mild (eg, infusion site inflammation and wheals) or severe (eg, desquamation, blister formation, and tissue necrosis) reactions depending on their mechanism of action.^1^^,^^2^

Our patient was treated with a combination treatment of pembrolizumab and enfortumab vedotin, which was recently approved for the treatment of locally advanced and metastatic urothelial carcinoma.^3^ Enfortumab vedotin is an antibody-drug conjugate of monomethyl auristatin E and a fully humanized IgG1 monoclonal antibody, directed at Nectin-4, an immunoglobulin-like adhesion protein highly expressed in urothelial carcinomas. Enfortumab vedotin is internalized after binding to Nectin-4, releasing monomethyl auristatin E into the cytoplasm and disrupting microtubule formation; this then leads to cell cycle arrest and apoptosis.^4^ Nectin-4 is also highly expressed in differentiating keratinocytes, suggesting that the cutaneous manifestations of enfortumab vedotin may be a consequence of direct binding of the antibody-drug conjugate to keratinocytes with immediate effects of cytotoxic monomethyl auristatin E.^5^

In the EV-301 trial (NCT03474107), which demonstrated significant survival benefit of enfortumab vedotin over single-agent taxane or vinflunine, any skin reaction occurred in 43.9% in patients. A total of 5.0% of all patients also experienced a severe cutaneous adverse reaction, grade 3 or higher, which includes drug eruption, bullous dermatitis, skin exfoliation, erythema multiforme, exfoliative rash, and pemphigus.^6^ Median time to onset of skin reaction was 0.43 (0.03, 12.68) months.^6^ Cutaneous reactions were common and presented most frequently within the first few doses of the first cycle.

**Fig 2:**
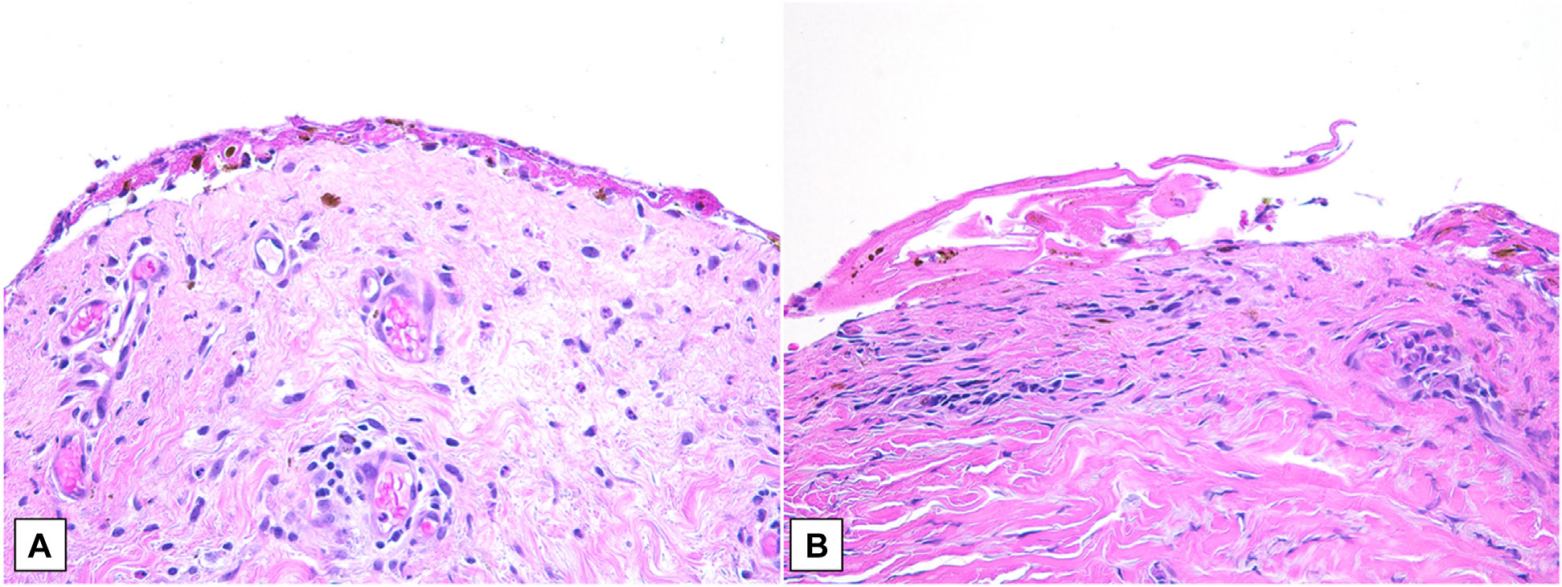
A, **B**, Biopsies revealing epidermal necrosis with subepidermal clefts.

In 1 patient in the EV-301 trial, biopsy of a skin reaction demonstrated colocalization of Nectin-4 and enfortumab vedotin in the skin with histopathologic features of vacuolar interface dermatitis, starburst mitotic figures, and maturation disarray of epidermal keratinocytes, consistent with the mechanism of action of enfortumab vedotin. In our patient, a biopsy revealed epidermal necrosis with subepidermal clefts (Fig 2, *A*, *B*). Although pembrolizumab has been associated with Stevens-Johnson syndrome and toxic epidermal necrolysis as well as other systemic inflammatory disorders, there is less documentation in the literature of significant extravasation reactions despite widespread use of the agent.

Prior local extravasation reactions to enfortumab vedotin have been observed and presented with erythematous plaque or bulla formation with central desquamation, erosion, and hemorrhagic crusting.^7^ This, taken with the findings in our patient, support the role of enfortumab vedotin as causing local epidermal necrosis if extravasated during infusion. The timing of onset of skin changes in relation to medication administration and the clinical extent of epidermal sloughing may raise concern among consulting providers for early evolving Stevens-Johnson syndrome/toxic epidermal necrolysis, which can be seen with systemic chemotherapy and other medications, although in this case the localization of the reaction to the site of medication administration and the absence of mucosal findings lower suspicion for Stevens-Johnson syndrome/toxic epidermal necrolysis.

Management of extravasation site reaction secondary to enfortumab vedotin includes stopping the infusion, attempting to aspirate the agent, removal of the catheter, and close monitoring of the reaction site. Dry compresses can encourage faster absorption of the agent. If signs of secondary infection develop, appropriate antibiotics should be initiated early. Prevention of site reactions can be achieved by ensuring appropriate placement of peripheral IVs before infusion. Additionally, using alternative central access, such as chemotherapy port placement, can prevent further local cutaneous reactions, although systemic reactions can still occur.

In this report, we describe a rare extravasation site reaction associated with administration of enfortumab vedotin, a chemotherapeutic agent known to cause cutaneous toxicity through a direct Nectin-4 mediated mechanism.

## Conflicts of interest

None disclosed.
